# Multiple facets of histone variant H2AX: a DNA double-strand-break marker with several biological functions

**DOI:** 10.1093/nar/gkv061

**Published:** 2015-02-20

**Authors:** Valentina Turinetto, Claudia Giachino

**Affiliations:** Department of Clinical and Biological Sciences, University of Turin, Orbassano, Turin, Italy

## Abstract

In the last decade, many papers highlighted that the histone variant H2AX and its phosphorylation on Ser 139 (γH2AX) cannot be simply considered a specific DNA double-strand-break (DSB) marker with a role restricted to the DNA damage response, but rather as a ‘protagonist’ in different scenarios. This review will present and discuss an up-to-date view regarding the ‘non-canonical’ H2AX roles, focusing in particular on possible functional and structural parts in contexts different from the canonical DNA DSB response. We will present aspects concerning sex chromosome inactivation in male germ cells, X inactivation in female somatic cells and mitosis, but will also focus on the more recent studies regarding embryonic and neural stem cell development, asymmetric sister chromosome segregation in stem cells and cellular senescence maintenance. We will discuss whether in these new contexts there might be a relation with the canonical DNA DSB signalling function that could justify γH2AX formation. The authors will emphasize that, just as H2AX phosphorylation signals chromatin alteration and serves the canonical function of recruiting DSB repair factors, so the modification of H2AX in contexts other than the DNA damage response may contribute towards creating a specific chromatin structure frame allowing ‘non-canonical’ functions to be carried out in different cell types.

## INTRODUCTION

In eukaryotes, DNA is structured into chromatin, an organization that is important for both resolving problems of spatial accommodation, and for functional utilization of the DNA and proper coordination of its metabolic activities (1,2). The monomeric building block of chromatin is the nucleosome, a flexible and dynamic structure (3,4) that contains ∼150 bp of DNA wrapped around a histone octamer consisting of two of each of the core histones H2A, H2B, H3 and H4 in 1.65 left-handed superhelical turns (5).

The replacement of canonical histones by histone variants (6) is one of the chromatin regulation mechanisms evolved by cells, influencing chromatin complexity by creating specialized nucleosomes. The H2A family contains a plethora of variants with some universal variants found in humans and other higher eukariotes, namely H2AX, H2AZ, macroH2A1, macroH2A2, H2A.F/Z and H2ABbd. The highest degree of diversification among histone H2A variants is generally in their C-termini, regarding both length and amino acid sequence (7,8).

The histone variant H2AX was first described in 1980 (9) and constitutes about 2.5–25% of total H2A in the mammalian genome (10). H2AX is defined by its SQ[E/D]Φ motif (where Φ is a hydrophobic amino acid) in the C-terminus. After DNA double strand breaks (DSBs), this serine (position 139 in humans) becomes phosphorylated (γH2AX) and renders H2AX an important player in preserving genome integrity.

**Figure 1.**
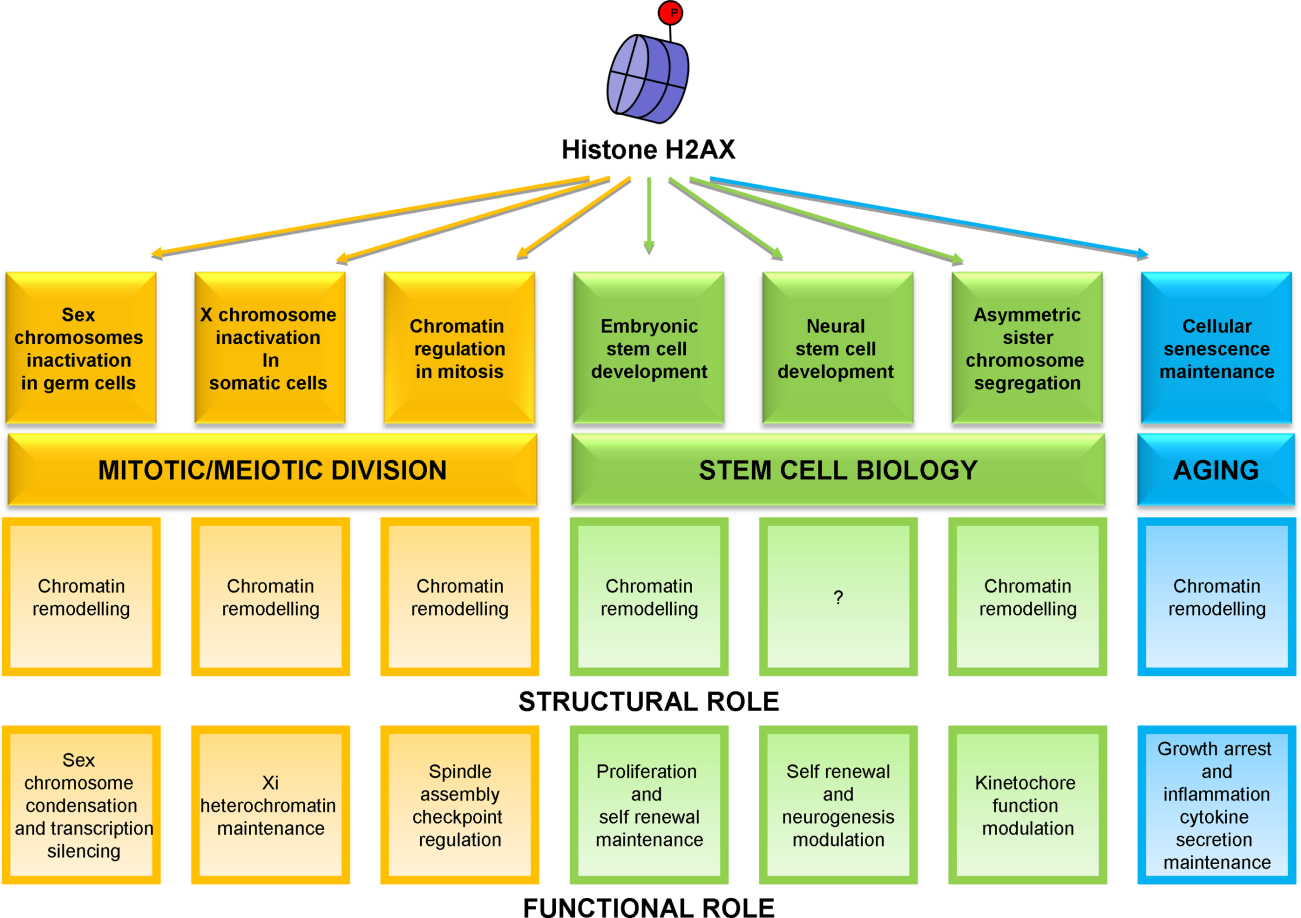
H2AX performs both structural and functional roles in the different non-canonical functions described beyond the DNA DSB response.

In the last decade, many works highlighted that H2AX and its phosphorylation on Ser 139 could not be simply considered as a specific DSB marker with a role restricted to the DNA damage response. Many reports presented H2AX as a ‘protagonist’ in other scenarios. In the following sections, we first briefly introduce the canonical H2AX role, then we present and discuss the up-to-date data regarding the ‘non-canonical’ ones (Table 1), focusing in particular on possible functional and structural roles capable to carry out specialized functions in different cell types (Figure 1). We will discuss how much the formation of γH2AX necessary to mediate these additional biological roles might be stimulated by the presence of DNA DSBs. Possibly in all the described biological processes the presence of either induced or naturally occurring DSBs promotes the initial H2AX phosphorylation; importantly, after this ‘priming’ H2AX becomes a protagonist of additional biological functions unrelated to the DNA DSB response.

**Table 1.** Overview of the up-to-now described histone H2AX non canonical roles, with references to the most relevant publications.

The histone H2AX *non-canonical* roles	First description	References
Sex chromosomes inactivation in germ cells	2002	(**28**)
		(30)
		(29)
		(35)
X chromosome inactivation in somatic cells	2005	(**42**)
		(39)
		(47)
Chromatin regulation in mitosis	2005	(**54**)
		(55)
		(58)
Neural stem cell development	2008	(**79**)
		(80)
		(81)
Cellular senescence maintenance	2009	(**97**)
		(96)
		(98)
		(106)
Embryonic stem cell development	2009	(**66**)
		(67)
		(72)
Asymmetric sister chromosome segregation	2013	(**83**)
		(85)

## THE HISTONE H2AX CANONICAL ROLE

After DSB occurrence, phosphorylation of H2AX on serine 139 results in the formation of γH2AX foci, which extend for up to 50 kb on each side of the DSBs in *Saccharomyces cerevisiae* (11) and for up to several Mb in mammals (12). H2AX phosphorylation is an early event in the DSB response leading to structural alterations at the damaged site to promote DNA repair. The conventional model for γH2AX focus formation suggests that after initiation near the break by ATM and/or DNA-PK (13), amplification occurs by spreading through the action of MDC1 binding to γH2AX (14). MDC1 in turn recruits the MRN complex (MRE11–RAD50–NBS1) (15) and the MRN complex further activates ATM (16). This generates a positive feedback loop to drive spreading of the phosphorylation away from the break.

H2AX^−/−^ cell lines display only moderate sensitivity to ionising radiation but fail to maintain DNA repair foci, suggesting that the crucial role of γH2AX is not direct recruitment of repair factors, but retention of these factors nearby the DSBs, hence preventing diffusion of the damaged ends away from each other (17,18).

Intrinsically, H2AX phosphorylation must take place within the context of chromatin structure. To allow an efficient repair, chromatin decondenses near the DSBs (19), but the mechanism for this remodelling is unclear. Serine 139 of H2AX is located near the DNA entry/exit point on the nucleosome, so one putative mechanism for the chromatin structural change is to be driven directly by the chemical properties of the added phosphate group. Although there are studies pointing towards a direct destabilization of the nucleosome by γH2AX (20,21), two reports in *S. cerevisiae*, using serine to glutamate mutants to mimic a phosphorylated serine, came to controversial results (22,23). As chromatin decondensation is not severely impaired in H2AX^−/−^ cells (18,19), it has been suggested that the critical role of γH2AX is the retention of remodelling factors at the repair site to define a damage neighborhood for efficient repair (24).

In conclusion, in the context of the DSB response H2AX appears to have both structural and functional roles, cooperating either directly or indirectly in chromatin decondensation and participating in the repair process through the retention of specific factors nearby the DSB.

## THE HISTONE H2AX NON-CANONICAL ROLES

### Sex chromosomes inactivation in germ cells

During meiotic prophase in male mammals, the X and Y chromosomes condense to form a macrochromatin body, termed sex body, within which X- and Y-linked genes are transcriptionally repressed. The molecular basis and biological function of both sex body formation and meiotic sex chromosome inactivation (MSCI) have long remained unknown. The condensation of the gonosomes to form a sex body is likely mediated by the association of specific proteins to the chromatin and by differential histone modifications (25–27). The observation that H2AX-deficient male, but not female, mice are infertile and have increased levels of X–Y asynapsis (28) was the first evidence that suggested a role of H2AX in MSCI. Subsequent studies showed that the X and Y chromosomes of histone H2AX-deficient spermatocytes fail to form a sex body, do not initiate MSCI, and exhibit severe defects in meiotic pairing. Moreover, other sex body proteins, including macroH2A1.2 and XMR, do not preferentially localize with the sex chromosomes in the absence of H2AX (29). Thus, H2AX is required for the chromatin remodelling and associated silencing in male meiosis.

Preparatory to description of this first non-canonical role was the observation that the phosphorylated form of H2AX localizes preferentially to the gonosomal chromatin in late zygotene/early pachytene spermatocytes, in a manner independent of meiotic recombination-associated SPO11-mediated DSBs (30). Analysis of H2AX distribution during male meiosis revealed that at zygotene the histone is widely and uniformly distributed throughout the autosomal chromatin whereas it is only modestly present in the gonosomal domain. In contrast to H2AX, an intense γH2AX signal develops over the gonosomal chromatin. This means that SPO11-independent γH2AX staining of gonosomal chromatin is due to a massive phosphorylation of H2AX, rather than a preferential increase in the local concentration of H2AX. The phosphorylated isoform subsequently disappears from the gonosomal chromatin precisely when the sex body disperses at the diplotene-metaphase I transition (30).

One interesting feature of the sex body-associated H2AX phosphorylation is its independence not only of meiotic recombination-associated DSBs mediated by SPO11 (30), but also of the ATM and DNA-PK kinases. Besides ATM and DNA-PK, the only other phosphatidylinositol 3-kinase like protein kinase (PI3KK) known to phosphorylate H2AX is ATR (31). Accordingly, a complete overlap in ATR and γH2AX staining in pachytene cells was reported (30,32–34). Additionally, in the normal testis BRCA1 and ATR interact either directly or indirectly within a protein complex and this interaction is important for the recruitment of ATR to the sex chromosomes (32). The timing of appearance and disappearance of BRCA1 on the sex chromosomes corresponds closely to that of γH2AX. These findings support a model in which BRCA1 has a role in recruiting ATR to the XY chromatin of pachytene spermatocytes where ATR phosphorylates H2AX that in turn triggers chromatin condensation and transcriptional repression.

A more recent work contributed to uncover the molecular mechanism underlying MSCI, focusing on MDC1 (35). Analysis on Mdc1^−/−^ spermatocytes revealed that MDC1 is an essential factor for spreading γH2AX from the axes to the chromosome-wide domain of sex chromosomes in the pachytene stage. These observations suggest that the state of H2AX phosphorylation regulates its ability to remodel chromatin in order to obtain silencing of the male sex chromosomes. According to this model, H2AX phosphorylation would be essential for initiation of heterochromatization in the sex body, and subsequent dephosphorylation of H2AX would be required for effecting the decrease in sex chromatin condensation at the end of diplotene, ensuring a proper chromosome configuration before metaphase I.

Notably, meiotic recombination in mice is initiated by programmed DSBs catalysed by the transesterase SPO11 (36) during leptotene (30). γH2AX forms at sites of SPO11-mediated DSBs, and, subsequently, the loss of γ-H2AX staining occurs in conjunction with synapsis (30). However, the location of γH2AX to the gonosomal chromatin domain in late zygotene is independent of SPO11-mediated DSBs. It is not yet known whether this SPO11-independent augmentation of the γH2AX signal is due to further DSB formation, but it is tempting to relate it to the condensation of the X and Y chromatin that occurs at the end of zygotene.

### X chromosome inactivation in somatic cells

X inactivation (Xi) is the process through which in mammalian females one of the two X chromosomes is inactivated to equalize the X-linked gene dosage between XX females and XY males. To perform Xi, placental mammals acquired a noncoding RNA (*Xist*) that decorates an entire X chromosome to initiate chromosome-wide gene silencing (37,38). The subsequent stability of the Xi is likely due to the faithful retention of the many overlapping epigenetic mechanisms of gene silencing acquired at the Xi including DNA methylation, nonrandom histone variant distribution, and a variety of core histone posttranslational modifications (39). An attractive period in which facultative heterochromatin can be maintained is during S-phase as the underlying DNA sequence is replicated (40).

H2AX was found neither enriched nor deficient at the Xi (41). However, in view of the crucial role of γH2AX in MSCI, a possible second, non canonical role in Xi process was hypothesized. Indeed, during early and mid S-phase, γH2AX displayed a speckled nuclear pattern, whereas in late S-phase, γH2AX levels were specifically increased within the territory of the Xi (42). BRCA1 was also found to transiently interact with the Xi chromatin in late S-phase during the period in which the Xi is replicated and this association was closely correlated with γH2AX (42). As BRCA1 associates with chromatin-remodelling machinery (43,44) and DNA helicases (45,46) possibly BRCA1 and γH2AX are involved in presenting the newly formed Xi chromatin in a favourable configuration for histone-modifying complexes to efficiently re-establish the specific combination of histone modifications present on Xi nucleosomes (39). A recent report described that the WSTF-ISWI chromatin remodelling complex (WICH) associates with the Xi during late S-phase as the Xi DNA is replicated. Elevated levels of WICH at the Xi is restricted to late S-phase and appears before BRCA1 and γH2AX. The sequential appearance of WICH and BRCA1/γH2AX implies each as performing important but distinct roles in the maturation and maintenance of heterochromatin at the Xi (47).

Given that BRCA1 and WICH are both implicated in maintaining heterochromatin (48,49) and that mouse Xi chromatin is compromised when the kinases responsible for H2AX serine 139 phosphorylation are inhibited (50), both direct and indirect roles in maintaining Xi heterochromatin are suggested.

Interestingly, γH2AX presence within the territory of the Xi in late S-phase follows an initial involvement of γH2AX in early and mid-S-phase, which appears to be related to spontaneous DSBs that occur throughout homologous recombination and sister chromatid exchange (51,52); H2AX phosphorylation within the territory of the Xi could be promoted by the previous H2AX activation mediated by DNA DSB signalling.

### Chromatin regulation in mitosis

Chromatin alterations occurring during mitosis not only are required for the condensation of chromosomes into discrete units, but also have functional roles, such as transcription factors displacement and maintenance of a memory of transcriptional programs after cell division. It has become clear in the past few years that post-translational modifications of histones play critical roles in both structural and functional chromatin regulation during mitosis (53).

In 2005, it was documented for the first time cell cycle dynamics of γH2AX foci in mammalian cells (54). The authors demonstrated that, without exogenous or artificial sources of DNA damage, hundreds of H2AX phosphorylated sites exist throughout the genome of normally growing mammalian cell lines. The existence of two distinct yet highly discernible γH2AX focal populations was documented: a first small population of large amorphous foci, morphologically similar to the ionizing irradiation-induced DSB foci, colocalizing with numerous DSB repair proteins and likely representing naturally occurring DSBs; and a second previously undescribed but much more abundant population of small foci, that do not associate with proteins involved in DNA DSB repair. Cell cycle analyses revealed that γH2AX signal intensities increased as cells progressed from G_1_ into S, G_2_ and M phase, reaching maximal levels at metaphase. Increased signal intensities occurred along the entire length of chromosome arms and were not excluded from highly compacted regions such as pericentromeric heterochromatin. Subsequently other studies contributed to describe γH2AX dynamics during the cell cycle, highlighting its specific presence during mitosis (55–57).

ATM null cell lines did not present the mitosis-specific increase in the γH2AX homogeneous staining of chromosomes observed when ATM was present within the cell (54,55), suggesting that ATM kinase activity is required during the initial stages of mitosis and responsible for most of the mitosis-specific increase in γH2AX levels.

These works were mainly centred on the description of mitosis-specific increase in γH2AX levels and identification of the main kinase involved in H2AX phosphorylation but did not define any functional roles for γH2AX during mitosis. γH2AX may contribute to the fidelity of the mitotic process, even in the absence of DNA damage, thereby ensuring the faithful transmission of genetic information from one generation to the next. A more recent work demonstrated for the first time a specific function of H2AX phophorylation during mitosis (58). The authors investigated for a possible role of DDR components in regulating mitotic spindle assembly checkpoint (SAC) activation, the process that verifies the correct kinetochore attachment during metaphase and regulates metaphase to anaphase transition by ensuring the fidelity of chromosome segregation (59). They demonstrated a mitotic role for MDC1 and ATM in regulating SAC activation and showed that H2AX is phosphorylated at mitotic kinetochores by ATM and this phosphorylation is needed for MDC1 localization at kinetochores and subsequent localization of MAD2 and CDC20 at kinetochores and for the formation of an intact mitotic checkpoint complex (58). This study could not distinguish whether the role of ATM, MDC1 and H2AX in SAC activation is due to a DNA damage-independent function of these DDR proteins or if SAC is activated by the DDR. In the first scenario, a novel role for these DDR proteins in the SAC, distinct from their DDR role, could be supposed. In the second scenario, ATM, MDC1 and H2AX will act in the SAC as DDR proteins, under the hypothesis that the SAC can also be activated by DNA damage. If so, possibly DSBs exist at centromeric regions during mitosis, as a result of endogenous processes.

### Embryonic stem cell development

During fertilization and pre-implantation development, the cells switch from terminally differentiated germ cells to undifferentiated totipotent zygotes and begin to differentiate at the late pre-implantation stage (60). During this period, the gene expression pattern changes substantially (61), as a consequence of genome-wide chromatin remodelling, which involves epigenetic reprogramming. In mammals, DNA methylation and histone modification are the two major epigenetic alterations.

A comprehensively histone variant gene expression profiling revealed that H2AX is among the histone variants highly expressed in undifferentiated mouse embryonic stem cells (mESCs) and in preimplantation embryos, from zigotene to blastocyst stage (62). Parallel studies on H2A species incorporation highlighted significant H2AX incorporation during the early pre-implantation stage. H2AX signal was detected after fertilization in both male and female pronuclei, the signal intensity remained high at the two- and four-cell stages, then it decreased (63). At variance only low levels of canonical H2A, H2A.Z and macroH2A were detected (63,64). Although Ser139 phophorylation appeared to be dispensable for H2AX incorporation (63) H2AX is highly phosphorylated throughout preimplantation development without any induced DNA damage (65). These results suggested that H2AX may have the potential of playing multiple roles during mouse embryogenesis.

In addition to these works describing H2AX abundance and distribution in embryonic stem cells, recent research has described potentially new and specialized roles for γH2AX in mESCs. The suggested roles focused on specialized functions characteristic of the embryonic state maintenance. Banath *et al*. for the first time associated high basal γH2AX levels in ESCs with global chromatin decondensation rather than pre-existing DNA damage (66). They described that mESCs express ∼100 large γH2AX foci per cell, that decrease during ESC differentiationand are not associated with any DDR factors, such as 53BP1 and RPA. They provided evidence for a redundant role of different kinases belonging to the PI3KK family (ATM, DNA-PK, and possibly ATR) in maintaining the high basal levels of H2AX phosphorylation observed in mESCs (66). Our group demonstrated that γH2AX epigenetic modification contributes to sustaining the self-renewal and proliferation ability of mouse ESCs and induced pluripotent stem cells (iPSCs) (67). In support to γH2AX role in mESCs self-renewal, γH2AX levels increased when ESCs were treated with small molecule inhibitors that enhance self-renewal and an H2AX^−/−^ ESC line had a reduced capacity for self-renewal that could be restored through reconstitution with wild-type H2AX, but not with a mutant form of H2AX in which the S139 phosphorylation site was abolished (67). Demonstration that high γH2AX levels sustain self-renewal of ESCs and iPSCs suggests this minor histone modification as another important epigenetic element involved in maintaining the chromatin architecture that contributes to the unique properties of pluripotent stem cells (68–71).

Recently, Wu *et al*. explored the new functions of H2AX in pluripotent stem cells (72) through a ChIP-seq approach aimed at analyzing the genome-wide deposition pattern of H2AX in mESCs. Strikingly, they found that ESC-specific H2AX deposition regions correlate with the silenced extraembryonic genes targeted by CDX2 in mESCs. They found that H2AX deficiency leads to upregulation of some extraembryonic genes, but not of pluripotency genes or germ layer markers. H2AX deposition thus seems critical in maintaining H3K9me3 level, but not H3K27me3, at extraembryonic gene enhancers. The authors also analysed the H2AX deposition pattern in different iPSC lines and determined their developmental potential by tetraploid complementation. Interestingly, the iPSC lines that were capable of tetraploid complementation and the ones that failed were classified into two distinct groups with an unsupervised hierarchical cluster analysis of H2AX deposition (72).

To support the role of H2AX in embryogenesis, H2AX presence during embryonic development was also documented in other species, including *Xenopus laevis* (73), porcine embryos (74) and different human ESC lines (75).

In discussing H2AX role in embryogenesis, it has to be taken in consideration that ESCs are rapidly dividing cells and it is thus possible to ascribe H2AX activation to the elevated replication stress. In support to this possibility, high levels of γH2AX have been associated with the single-strand breaks occurring in S-phase, and ESC populations have an increased proportion of cells in S-phase compared to many somatic cells (76).

### Neural stem cell development

Recent studies on neural stem cells (NSCs) described a H2AX-dependent pathway able to modulate NSC self-renewal, niche size and neurogenesis in adult brain. GABA_A_ receptor-mediated events act to regulate neural stem cell proliferation both in the developing cortex (77) and in the adult subventricular zone (SVZ) (78). Through a variety of *in vitro* and *in vivo* studies, Andäng *et al*. demonstrated that mouse neural crest stem cells express glutamic acid decarboxylase and functional GABA_A_ receptors and showed that endogenously produced GABA acts through ATM/ATR PI3KK-family proteins and regulate NSC proliferation; interestingly, the effect of GABA on cell proliferation was critically dependent on H2AX (79). Then, they studied how GABA signalling regulates adult neurogenesis in SVZ stem cell niche and demonstrated that the number of dividing cells was markedly affected in SVZ clones by GABA_A_R signalling following acute treatment, such that muscimol (a GABA_A_R agonist) decreased and bicuculline (a GABA_A_R antagonist) increased numbers of dividing cells. This effect was dependent on H2AX, as it was completely abolished in H2AX-deficient cells. These *in vitro* studies demonstrated that NSC numbers can be modulated in both directions by GABA_A_R signalling in a manner dependent on the presence of histone H2AX (80).

To sustain a specific role of γH2AX in neural stem cell development, a map was produced on the occurrence and distribution of γH2AX in developing, postnatal, adult, and senescent mice along the entire rostro-caudal axis of the brain by coronal serial sectioning and immunocytochemistry at light microscopy level (81). H2AX was shown to be phosphorylated during embryonic, postnatal and adult neurogenesis in all areas of the intact mouse brain that display and/or retain proliferative capacity, primarily the SVZ/rostral migratory stream/olfactory bulb system and the cerebellar cortex. γH2AX in interkinetic nuclei was mainly observed to occur in foci, albeit diffuse staining of the nucleoplasm was also evident. The observation of a connection between neural cell proliferation capacity and presence of γH2AX suggests that also during neural stem cell development DNA damage occurring as a consequence of replicative stress might somehow promote H2AX phosphorylation.

Differently from the other H2AX non-canonical functions, a possible structural H2AX role in this field remains to be determined and could be an interesting matter for future studies.

### Asymmetric sister chromosome segregation

In 1975, John Cairns proposed the ‘immortal strand’ hypothesis claiming that ‘stem cells would be protected against errors of duplication if it were so arranged that the immortal daughter cell always receives the DNA molecules which have the older of the two parental strands and the mortal daughter always collects the molecules with the younger parental strand’ (82). Recently Charville and Rando revised this model by proposing the ‘mortal strand’ hypothesis (83). They hypothesized that the segregation of sister chromatids according to relative template strand age is a consequence of replication stress, that is defined as ‘inefficient DNA replication that causes DNA replication forks to progress slowly or stall’ (84). In their model, replication stress generates asymmetric DNA damage, that focus on chromosomes bearing newer template DNA. This creates a situation in which it is advantageous to preferentially segregate chromosomes bearing newer template strands, in which there is DNA damage, to a single daughter cell. They hypothesize that the generation of asymmetric DNA damage on sister chromatids by replication stress could initiate a local epigenetic response that serves as a mark of sister chromatids bearing newer template DNA strands. In this model they proposed a specific role of H2AX phophorylation, suggesting that the DSBs generated by DNA replication on the newer template strand would recruit DNA damage response factors, including PI3KK family members ATM/ATR, which in turn would phosphorylate H2AX. They speculate that γH2AX could function as a mark that distinguishes sister chromatids with newer template strand from those having the older template strand and could transmit a signal interpreted by the mitotic spindle as ‘pick me’ or ‘do not pick me’(83).

Preparatory studies conducted by Haber *et al*. using an inducible DSB system had demonstrated that one single DSB could delay mitosis by a γH2AX-dependent mechanism. Their findings provided evidence that DNA breaks generate a signal that is conveyed to the centromere of the chromosome on which the break occurred and that is ultimately communicated to the mitotic spindle (85). In their proposed model γH2AX, once at the centromere, impairs kinetochore function either directly, by distorting chromatin structure, or indirectly, by recruiting other factors that inhibit kinetochore formation. Owing to their age-related asymmetry and biased segregation in stem cells (86,87), centrosomes had already received attention for their possible role in non-random chromosome segregation (88). In *Drosophila* larval brain neuroblasts, a model system for studying asymmetric stem cell division, the two centrioles separate during interphase and distinguish each other for microtubule aster organization ability (89,90). The dominant centrosome remains stationary at the cell's apical cortex, goes on to form one pole of the mitotic spindle and never loses microtubule-nucleating activity. In contrast, the other centriole moves extensively throughout the cell during interphase and loses microtubule-nucleating activity. The dominant centriole is generally inherited by the self-renewing stem cell, whereas the more motile centriole is segregated to the differentiating daughter cell. In the mortal strand hypothesis, the different centrosome microtubule-organizing capabilities could account for the coordinated inheritance of sister chromatids with DNA damage: the newer template strands may contain factors possibly recruited by γH2AX (or other DDR proteins) that discourage astral microtubule attachment, favouring the dominant centrosome attachment to the undamaged sister chromatids.

In this model, the initial activation of H2AX is promoted by DNA damage that occurs during stem cell division and subsequently γH2AX will function as a marker that directs chromosome segregation.

### Cellular senescence maintenance

Cellular senescence is a condition initially described by Hayflick in human fibroblasts as a state of permanent cell cycle arrest resulting from serial passage in culture due to a limited proliferative lifespan (91). Cellular senescence limits the proliferation of damaged cells at risk for neoplastic transformation by imposing an essentially irreversible growth arrest. Senescent cells undergo distinctive changes (92–95) including the accumulation of persistent γH2AX foci, differing, both in size and persistence, from the transient foci that occur during initial successful DSB rejoining. When they were described for the first time, they were defined as ‘cryptogenic γ-foci’ and, considering the almost complete co-localization between the γ-foci and repair proteins 53BP1, MRE11, RAD50 and NBS1, they were considered γ-foci containing unrepairable DSBs (96). However, in 2009, it was published the first work describing the presence of Persistent DNA Damage Foci (PDDF) in senescent fibroblasts as an event that may occur without detectable DNA damage, as a result of the typical DDR activation reported in senescent cells (97). The authors demonstrated that PDDF, containing γH2AX foci and persistent DDR signalling, can independently control at least two important phenotypes: the p53-dependent senescent growth arrest and the p53-independent senescence-associated extracellular inflammatory signalling. Subsequently, the same authors carefully characterized PDDF in fibroblasts identifying sequential events that differentiate transient from persistent foci, which they term ‘DNA segments with chromatin alterations reinforcing senescence’ (DNA-SCARS) (98). DNA-SCARS contain many proteins also present in transient foci or foci that mark dysfunctional telomeres (12,99–103), such as γH2AX, the repair or adaptor proteins MDC1, NBS1, MRE11, ATM-pSer-1981 and CHK2-pThr68; however, persistent damage foci lack evidence of the active DNA repair that occurs in transient foci, supporting the idea that persistent damage foci are not sites of replicative or repair DNA synthesis, but instead are stable chromatin alterations. H2AX depletion did not interfere with 53BP1 foci formation; differently the DDR adaptor protein MDC1 and CHK2-pThr68 were either absent or reduced in H2AX-depleted 53BP1 foci. Thus, H2AX deficiency interferes with the efficient assembly of some DDR proteins into DNA-SCARS. Interestingly, H2AX depletion suppressed the DDR-dependent senescence-associated growth arrest and IL-6 secretion, indicating that the activated DDR signalling during senescence has a functional role, mediated also by γH2AX presence. Supporting the idea that senescent PDDF are a signature of stable chromatin alterations, rather than classical DSBs, changes in chromatin organization are sufficient to induce senescence and are associated with increase in H2AX phosphorylation (104,105). Recently our group described for the first time the presence of PDDF in adult stem cells (106), thus extending the impact of this non-canonical H2AX role in senescence maintenance to undifferentiated cells.

One interesting possibility is that DNA-SCARS provide a reservoir for active DDR signalling, essential to maintain both the p53-dependent growth arrest and inflammatory cytokines secretion (97,99,100,107–109). A deeper understanding of how these structures assemble and function will probably enrich insights into the mechanisms that link DNA damage, inflammation, and aging.

As for the other described non canonical γH2AX functions, also in this case, it is possible to suppose that damaged cells initially activate a canonical DDR response, including γH2AX foci formation; subsequently some DDR components, including γH2AX, will carry on specific activities able to sustain the senescence response.

## CONCLUSIONS

Many recent data support the importance of the histone variant H2AX in a multitude of biological processes including specific aspects of cell division, stem cell biology and aging (Figure 1). It appears clear that the presence of the phosphorylated form of H2AX influences the chromatin structure, determining a chromatin frame that allows to serve specific biological functions. Probably the cell type, the context in which this chromatin remodelling occurs and the presence of other remodelling complexes will all contribute to determine which biological function H2AX can promote. The contribution to many specialized functions from embryonic development to aging highlights the fundamental role of H2AX beyond the canonical DNA DSB response. Definition of these new functions in more details will surely deserve interesting scenarios.
